# Omentin-1 ameliorates the progress of osteoarthritis by promoting IL-4-dependent anti-inflammatory responses and M2 macrophage polarization

**DOI:** 10.7150/ijbs.86701

**Published:** 2023-10-16

**Authors:** Chih-Yuan Ko, Yen-You Lin, David Achudhan, Jun-Way Chang, Shan-Chi Liu, Chao-Yang Lai, Yuan-Li Huang, Chun-Hao Tsai, Yi-Chin Fong, Hsien-Te Chen, Kun-Tsan Lee, Chien-Chung Huang, Ting-Kuo Chang, Chih-Hsin Tang

**Affiliations:** 1 Graduate Institute of Biomedical Sciences, China Medical University, Taichung, Taiwan.; 2 Department of Orthopedic Surgery, China Medical University Hospital, Taichung, Taiwan.; 3 Department of Pharmacology, School of Medicine, China Medical University, Taichung, Taiwan.; 4 Program of Biotechnology and Biomedical Industry, China Medical University, Taichung, Taiwan.; 5 Department of Medical Education and Research, China Medical University Beigang Hospital, Yunlin County, Taiwan.; 6 Institute of Biomedical Sciences, Mackay Medical College, New Taipei City, Taiwan.; 7 Department of Medical Laboratory Science and Biotechnology, College of Medical and Health Science, Asia University, Taichung, Taiwan.; 8 Department of Sports Medicine, College of Health Care, China Medical University, Taichung, Taiwan.; 9 Department of Orthopedic Surgery, China Medical University Beigang Hospital, Yunlin, Taiwan.; 10 Department of Post-Baccalaureate medicine, National Chung-Hsing University, Taichung, Taiwan.; 11 Department of Orthopedics, Taichung Veterans General Hospital, Taichung, Taiwan.; 12 School of Medicine, China Medical University, Taichung, Taiwan.; 13 Division of Immunology and Rheumatology, Department of Internal Medicine, China Medical University Hospital, Taichung, Taiwan.; 14 Department of Medicine, Mackay Medical College, New Taipei, Taiwan.; 15 Division of Spine Surgery, Department of Orthopedic Surgery, MacKay Memorial Hospital, New Taipei, Taiwan.; 16 Chinese Medicine Research Center, China Medical University, Taichung, Taiwan.; 17 Department of Medical Research, China Medical University Hsinchu Hospital, Hsinchu, Taiwan.

**Keywords:** Omentin-1, Osteoarthritis, IL-4, M2 macrophage, Anti-inflammation

## Abstract

Osteoarthritis (OA) is a prevalent joint disease commonly associated with aging and obesity, which can lead to pain, stiffness, joint dysfunction, and disability. Omentin-1 (also called intelectin-1) is a newly discovered adipokine, which plays a protective role in suppressing the secretion of pro-inflammatory cytokines. Based on data from the Gene Expression Omnibus (GEO) dataset and clinical samples obtained at our institution revealed, determined that omentin-1 and IL-4 (an anti-inflammatory cytokine) levels were significantly lower in OA patients than in normal controls. Omentin-1 was shown to induce IL-4-depedent anti-inflammatory responses and M2 macrophage polarization in OA synovial fibroblasts via the PI3K, ERK, and AMPK pathways. Administering omentin-1 was shown to block cartilage degradation and bone erosion resulting from anterior cruciate ligament transection by inhibiting the production of pro-inflammatory cytokines and promoting M2 macrophage polarization *in vivo*. Our findings indicate omentin-1 as a promising therapeutic avenue for the treatment for OA.

## Introduction

Osteoarthritis (OA) is a prevalent joint disease commonly associated with aging and obesity 1, which can lead to pain, stiffness, and restricted joint mobility 2. The various manifestations of OA reflect a complex array of pathological mechanisms, including the activation of OA synovial fibroblasts (OASFs), cartilage degeneration, and the release of inflammatory cytokines 3. Despite ongoing research, current disease-modifying treatments have had little success in managing OA due to its complex pathogenesis 4.

Researchers have reported a positive correlation between synovial inflammation and progressive joint failure 5, attributable to proliferative and proinflammatory responses stimulated by the adjacent synovium, which often leads to the degradation of matrix products 3. Interleukin-1 beta (IL-1β), IL-6, IL-8 and tumor necrosis factor alpha (TNF-α) are key proinflammatory cytokines involved in the pathophysiology of OA 6. The concentration of these compounds in synovial tissue varies with OA grade 7. The catabolic effects of proinflammatory cytokines on OASFs can be observed in the strong induction of matrix metalloproteases (MMPs) and a disintegrin and metalloprotease with thrombospondin type I motifs (ADAMTS) 8. The anti-inflammatory cytokines primarily involved in the pathogenesis of OA include IL-4, IL-10, and IL-13 9. Previous research has indicated that IL-4 reduces TNF-α, IL-1β, and IL-6 levels by reducing lipopolysaccharide (LPS) and interferon gamma (IFN-γ) levels in the inflammatory phase 10. IL-4 also has an inhibitory effect on the degradation of proteoglycans in articular cartilage, by repressing the secretion of MMPs and mediating the production of proteoglycans that appear during the course of OA 10. Increasing IL-4 levels is a novel approach to the treatment of OA.

Macrophages are among the most abundant immune cells in the inflammatory synovium of OA patients, and are considered important contributors to the production of cytokines in OA 11. Macrophages in the OA joint become polarized into either the M1 or M2 phenotype under stimulation from inflammatory cytokines 12. Pro-inflammatory cytokines, such as TNF-α, IL-1β, and IFN-γ, enhance M1 polarization and lead to the expression of inflammatory cytokines, subchondral bone remodeling, and osteoclast generation in OA 12-14. Conversely, anti-inflammatory cytokines, such IL-4 and IL-13, promote M2 polarization and possess tissue-repair functions 15-17. These results indicate that controlling M2 polarization should be a critical objective in anti-OA therapy.

Numerous studies have demonstrated the regulatory effects of adipokines, including adiponectin, leptin, and visfatin, on the progression of OA 18. Omentin-1 (also called intelectin-1) is a newly discovered adipokine comprising 313 amino acids 19 mainly expressed in human omental and subcutaneous adipose tissue and the small intestine. It been shown to have a protective role in suppressing the secretion of IL-1β, IL-6, and TNF-α 20. Previous experimental studies have reported a positive correlation between omentin-1 and elevated levels of anti-inflammatory cytokines, such as IL-4 and IL-13 21; however, the role of omentin-1 in OA has yet to be elucidated. In the current study, we determined that the expression levels of omentin-1 and IL-4 are higher in normal individuals than in OA patients. By promoting IL-4 production, omentin-1 facilitates anti-inflammatory responses and M2 macrophage polarization via the phosphoinositide 3-kinase (PI3K), extracellular signal-regulated kinases (ERK), and 5'AMP-activated protein kinase (AMPK) signaling pathways. Administering omentin-1 was shown to block cartilage degradation and bone erosion resulting from anterior cruciate ligament transection by inhibiting the production of pro-inflammatory cytokines and promoting M2 macrophage polarization *in vivo*. These results provide new insights into the negative effects of omentin-1 on OA pathology resulting from the promotion of IL-4 production.

## Material and Methods

P-AMPK (SC-33524), AMPK (SC-25792), p-PI3K (SC-129289), p-PI3K (SC-1637), p-ERK (SC-7383), ERK (SC-1647) were purchased from Santa Cruz Biotechnology, Inc. (final dilution of 1:1000 for western blotting, Santa Cruz, CA, USA). Omentin-1 (A7234), IL-4 (A4988), TNF-α (A11534), Cluster of Differentiation 68 (CD68) (GTX41865), CD86 (GTX34569), and CD163 (NBP2-36495) were purchased from ABclonal, Inc., GeneTex, Inc. and Novus Biologicals (final dilution of 1:100 for Immunohistochemistry staining or Immunofluorescence staining). PI3K, AMPK, ERK, IL-4, and control ON-TARGET plus siRNAs were purchased from Dharmacon, Inc. (Lafayette, CO, USA). The qPCR primers and the Taqman ® one-step PCR Master Mix were supplied by Applied Biosystems (Foster City, CA, USA). Pharmacological inhibitors for PI3K (LY294002, 1 μM, catalog number: 440202; Wortmannin, 1 μM, catalog number: W1628), AMPK (Ara A, 1 μM, catalog number: P5499; Compound C, 1 μM, catalog number: 171260), ERK (ERK II, 1 μM, catalog number: 328007) and all other chemicals not previously mentioned were provided by Sigma-Aldrich (St. Louis, MO, USA).

### Bioinformatics analysis

We obtained data related to pro-inflammatory and anti-inflammatory cytokines in synovial tissue from normal healthy controls and OA patients from the Gene Expression Omnibus (GEO) database (https://www.ncbi.nlm.nih.gov/geo/; Reference Series GSE82107 and GSE29746) for analysis.

### Patients and clinical samples

Synovial tissue was obtained from 29 patients, which included 9 cases of joint injury requiring joint repair and 20 cases of radiographically-detected stage IV OA (based on Ahlbäck criteria) scheduled for knee replacement surgery. The suprapatellar pouch of the knee was the source of synovial tissue used for OASFs cultures and omentin-1, IL-4, CD68, CD86, and CD163 staining. All patients were treated at the China Medical University Hospital, Taichung, Taiwan. All patients provided written informed consent prior to participation in the study. All procedures were conducted in accordance with the Institutional Review Board (IRB) regulations and guidelines established by the IRB of China Medical University Hospital, Taichung, Taiwan.

### Cell cultures

OASF isolation via collagenase treatment involved culturing OASFs in Dulbecco's Modified Eagle Medium (DMEM; Invitrogen) supplemented with 10% (v/v) fetal calf serum (FCS) containing 50 U/L penicillin and 50 µg/mL streptomycin and glutamine under a humidified atmosphere with 5% CO_2_ in accordance with the methods outlined in 22. OASFs were seeded in a 10-cm dish at a plating density of 5 x 10^5^ cell/ml. In every experiment, analysis was performed on OASFs from passages 3-7. THP-1, a human leukemia cell line of monocyte/macrophage lineage, was obtained from American Type Culture Collection (Manassas, VA, USA) and grown in RPMI-1640 medium with 10% FBS. THP-1 monocytes were differentiated into macrophages via incubation with 20 ng/ml phorbol 12-myristate 13-acetate for 24 h followed by incubation in RPMI medium for 24 h. Macrophages were polarized into M1 macrophages via incubation with 20 ng/ml of IFN-γ and 10 pg/ml of LPS, and into M2 macrophages via incubation with 20 ng/ml of IL-4 and 20 ng/ml of IL-13. The characterization of labeled THP-1 cells was performed using a FACSCalibur^TM^ flow cytometer in conjunction with CellQuest^TM^ software (both from BD Biosciences, San Jose, CA, USA).

### siRNA transfection

The transfections of PI3K, AMPK, ERK, IL-4, and control siRNA were performed using Lipofectamine 2000 (Invitrogen) over a period of 24 hours, in accordance with the manufacturer's instructions 22.

### Real-time quantitative polymerase chain reaction amplification

RNA was extracted from OASFs using the TRIzol kit (MDBio, Taipei, Taiwan) in accordance with the protocol outlined by the manufacturer. The quality and quantity of RNA were determined using a Nanovue^TM^ Spectrophotometer (GE Healthcare, WI, USA) via A260 measurements. cDNA synthesis was performed using the M-MLV RT kit (Invitrogen, CA, USA) with 1 μg of total RNA in accordance with the manufacturer's instructions. Real-time quantitative polymerase chain reaction (qPCR) analysis was performed using the KAPA SYBR® FAST qPCR Kit (Applied Biosystems, CA, USA). The primer sequences are presented in Supplementary Table S1 23.

### Western blot analysis

Extracted proteins (loading amount: 30 μg) were resolved using SDS-PAGE and then transferred to PVDF membranes in accordance with methods detailed in our previous reports 24, 25. Briefly, the membranes were blocked in phosphate buffered saline with Tween20 (PBST) containing 4% non-fat milk for 1 h and then incubated with antibodies targeting p-AMPK, AMPK, p-PI3K, PI3K, p-ERK, and ERK for 1 h, followed by incubation with HRP-conjugated secondary antibodies for 1 h. The blot membranes were visualized using a Fujifilm LAS-3000 imaging system.

### Experiment: OA Model

Male Sprague-Dawley (SD) rats (8 weeks of age; 300-350 g) were obtained from the National Laboratory Animal Center in Taiwan and maintained in accordance with the Guidelines of the Animal Care Committee of China Medical University, Taichung, Taiwan. The rat model involved anterior cruciate ligament transection (ACLT) using the protocols outlined in our previous reports 26. Briefly, after preparing the right knee in a surgically sterile manner, ACL fibers were transected using a scalpel, followed by excision of the entire medial meniscus via a medial parapatellar mini-arthrotomy. Following surgery (day 0), the rats were divided into three groups (n=8 per group) including a control group, ACLT group, and ACLT plus omentin-1 (300 ng/50 μL saline) group. The ACLT plus omentin-1 group received intra-articular injections of omentin-1 every day for 6 weeks. All rats were allowed to move freely in plastic cages until necropsy at 10 weeks post-surgery.

### Dynamic weight bearing test

Weekly assessments of spontaneous pain and postural deficits were performed using the static weight-bearing Incapacitance Test (Bioseb, Paris, France). This involved placing the rats in an angled plastic chamber with the hind paws positioned on separate sensors to measure dynamic weight bearing between the limbs over a 10-second period. The results are expressed in grams. The force value was obtained by subtracting the weight on the right limb from the weight on the left limb using the following formula: [Force = weight on left limb - weight on right limb]. Each experiment was performed three times, with the mean value recorded for each rat.

### Micro-CT scanning and analysis

Micro-CT scanning and analysis involved fixing femurs and tibias in 10% neutral formalin. Knee joint imaging was performed using an *in vivo* micro-CT scanner (Bruker SkyScan 2211 nano-CT, Bruker micro-CT, Kontich, Belgium) with the following scanner settings: 90 kVp voltage of X-ray source, 450 μA of current (8 watts output), and a 0.5 mm thick aluminum (Al) filter to reduce beam-hardening artifacts. InstaRecon® software (Bruker-micro-CT, Kontich, Belgium) was used for image reconstruction, the correction of ring artifacts, and beam-hardening. The reconstructed cross-sections were re-oriented and 59 slices (0.5mm) were selected. Regions of interest (ROIs) were selected based on the subchondral trabecular bone region of the medial tibial plateau in accordance with a methodology outlined in previous studies. The CTAn software (Version 1.7.1, Bruker microCT, Kontich, Belgium) was used to analyze the selected ROI for bone morphometry and bone mineral density.

### Histological analysis

Histological analysis involved fixing animal specimens in 10% neutral formalin for at least 72 h, followed by decalcification using 10% ethylenediaminetetraacetic acid in phosphate-buffered saline for 14 days, dehydration with increasing concentrations of ethanol, and embedding in paraffin. Section slides along the sagittal plane were then prepared using hematoxylin and eosin (H&E) or Safranin-O/Fast-green, in accordance with previously published procedures 26. In Osteoarthritis Research Society International (OARSI) histopathology evaluating system, the grade of cartilage damage is defined from 0 to 6 as the depth of progression of OA into the cartilage, and the horizontal extent of cartilage involvement is defined from 0 to 4. The final score is from 0-24 (grade 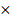 stage). The score of synovium inflammation is from 0-5 (Score=0, normal; Score=1, less than 3-4 lining cell layers or slight proliferation of synovial tissue; Score=2, 3-4 lining cell layers and proliferation of synovial tissue; Score=3, more than 4 lining cell layers and proliferation plus infiltration of subsynovial tissue with inflammatory cells; Score=4, more than 4 lining cell layers and proliferation plus infiltration of synovial tissue with a large number of inflammatory cells).

### Immunohistochemistry (IHC) staining

Human synovial tissues and rat joint sections from the *in vivo* ACLT model were stained using anti-omentin-1, IL-1β, IL-4, IL-6, IL-8, and TNF-α antibodies. Staining results were quantified using methods described in our previous study 27. In brief, 5 μm tissue section slides underwent dewaxing with xylene followed by rehydration using ethanol (100% to 75%). Subsequently, they were immersed in 3% hydrogen peroxide to block endogenous peroxidase activity for 10 minutes at 25°C. After trypsinization, the sections were further blocked with 4% bovine serum albumin in PBS for 30 minutes at 25°C. Next, the sections were incubated overnight at 4°C with primary antibodies (anti-omentin-1, IL-1β, IL-4, IL-6, IL-8, and TNF-α). The secondary antibody and DAB staining were performed using the Leica Novolink Polymer Detection Systems (Leica Biosystems Inc, IL, US). The final staining scores (ranging from 0 to 7) were obtained by adding the intensity and percentage scores 28.

### Immunofluorescence staining

Human and rat tissue sections were stained using anti-CD68, CD86, and CD163 antibodies in accordance based on an established protocol 29. In brief, 5 μm tissue section slides were subjected to wax removal using xylene, followed by rehydration with ethanol (100% to 75%) and trypsinization. The sections were then blocked with 3% bovine serum albumin in PBS for 30 minutes at 25°C. Next, the tissues were incubated overnight at 4°C with a mixture of primary antibodies (anti-CD68, CD86, and CD163). Subsequently, a mixture of secondary antibodies, including Alexa Fluor 488-conjugated chicken anti-mouse IgG antibody, Alexa Fluor 594-conjugated goat anti-rabbit IgG antibody, and Alexa Fluor 647-conjugated goat anti-rat antibody, was applied for 60 minutes at 25°C while avoiding exposure to light. The percentage of CD68/CD86 or CD68/CD163 was determined and quantified using TissueQuest software after scanning the sections with the TissueFaxs Spectra (Vienna, Austria).

### Statistical Analysis

For all quantified results, the mean ± SD of at least 3 experiments was calculated using GraphPad Prism 5.0 software. The student's t-test was used for statistical comparison of 2 groups, whereas two-factor analysis of variance (ANOVA) with Bonferroni *post hoc* test or the Mann-Whitney U test was used for statistical comparisons of more than 2 groups, as appropriate. In all cases, statistical significance was determined as a *p*-value of less than 0.05.

## Results

### Omentin-1 and IL-4 are negatively correlated with OA progression

**Figure 1:**
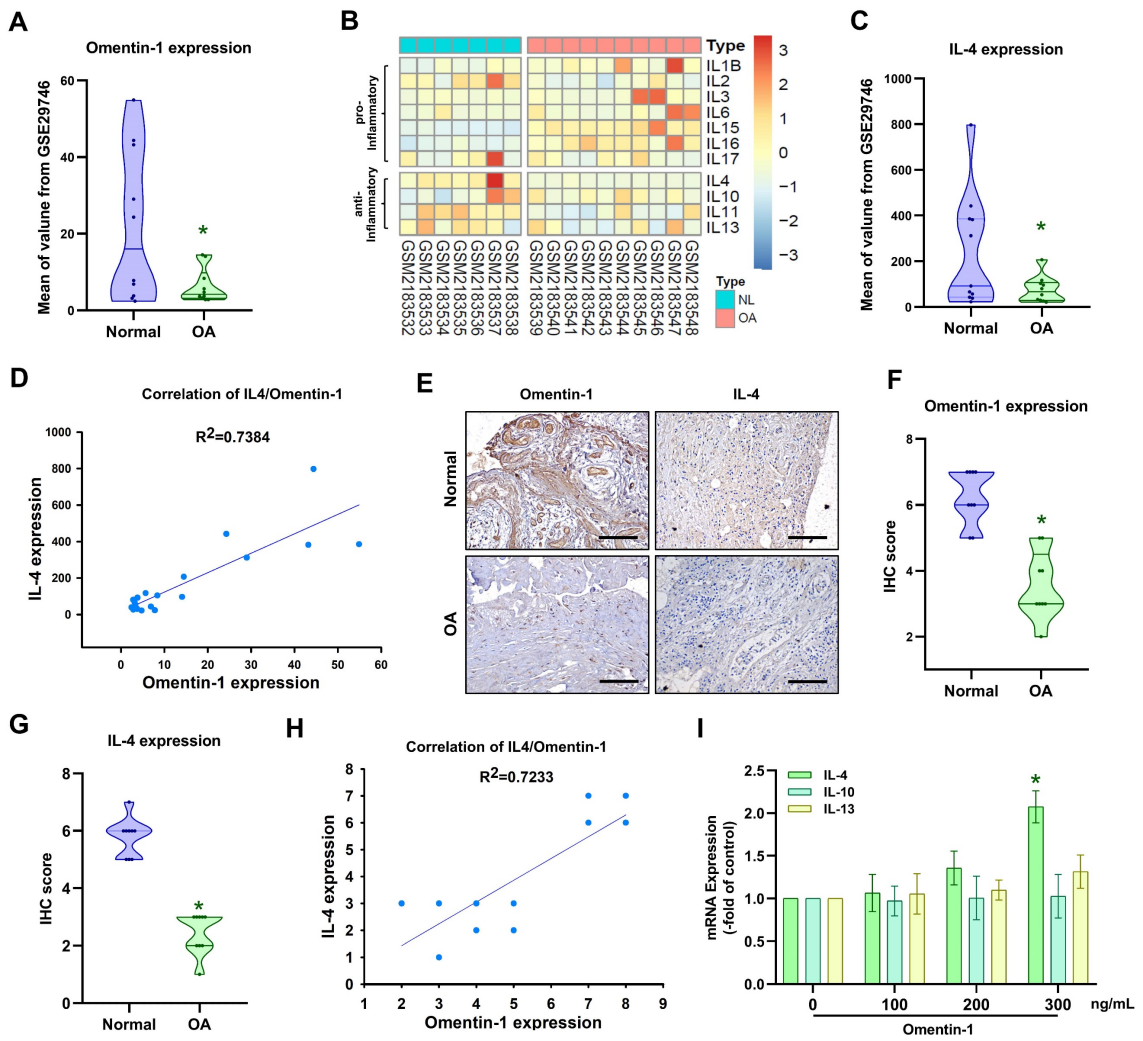
** Omentin-1 and IL-4 expression levels are negatively correlated with OA progression.** (A) Omentin-1 levels and (C) IL-4 levels in normal and OA synovial tissue samples retrieved from the GEO dataset; (B) Heatmap showing differentially expressed IL family genes from the GSE82107 dataset; (D) Positive correlation between omentin-1 and IL-4 based on the GSE29746 dataset; (E-H) IHC staining showing that the levels of omentin-1 and IL-4 were higher in human OA synovial tissue than in healthy tissue. Scale bar = 100 µm. (I) qPCR analysis showing that treating OASFs with omentin-1 increased IL family gene expression. *n*=6 for each group. * *p*<0.05 vs controls.

Adipokines are reported to play a key role in the development of OA 30; however, the expression of adipokine omentin-1 in OA is largely a mystery. Our analysis of the GEO dataset revealed that omentin-1 levels are significantly lower in OA patients than in normal controls (Fig. 1A). The balance between pro-inflammatory and anti-inflammatory IL family cytokines is crucial to OA progression 31. We consulted the GEO database pertaining to the expression of pro- and anti-inflammatory IL cytokines in normal (i.e., healthy) and OA synovia (Fig. 1B). Among the anti-inflammatory IL cytokines, only IL-4 (i.e., not IL-10, IL-11, or IL-13) expression was significantly higher in normal individuals than OA patients (Fig. 1B and C). Linear regression analysis revealed a significantly positive correlation between the expression levels of omentin-1 and IL-4 (Fig. 1D). IHC analysis of our clinical samples also confirmed that omentin-1 and IL-4 were more strongly expressed in normal controls than in OA individuals (Fig. 1E-H). Stimulating OASFs with omentin-1 increased IL-4 expression (but not IL-10 or IL-13) in a concentration dependent manner (Fig. 1I), which indicates that omentin-1 and IL-4 play contradictory roles in OA development.

### Omentin-1 promotes IL-4-dependent anti-inflammatory effects

**Figure 2:**
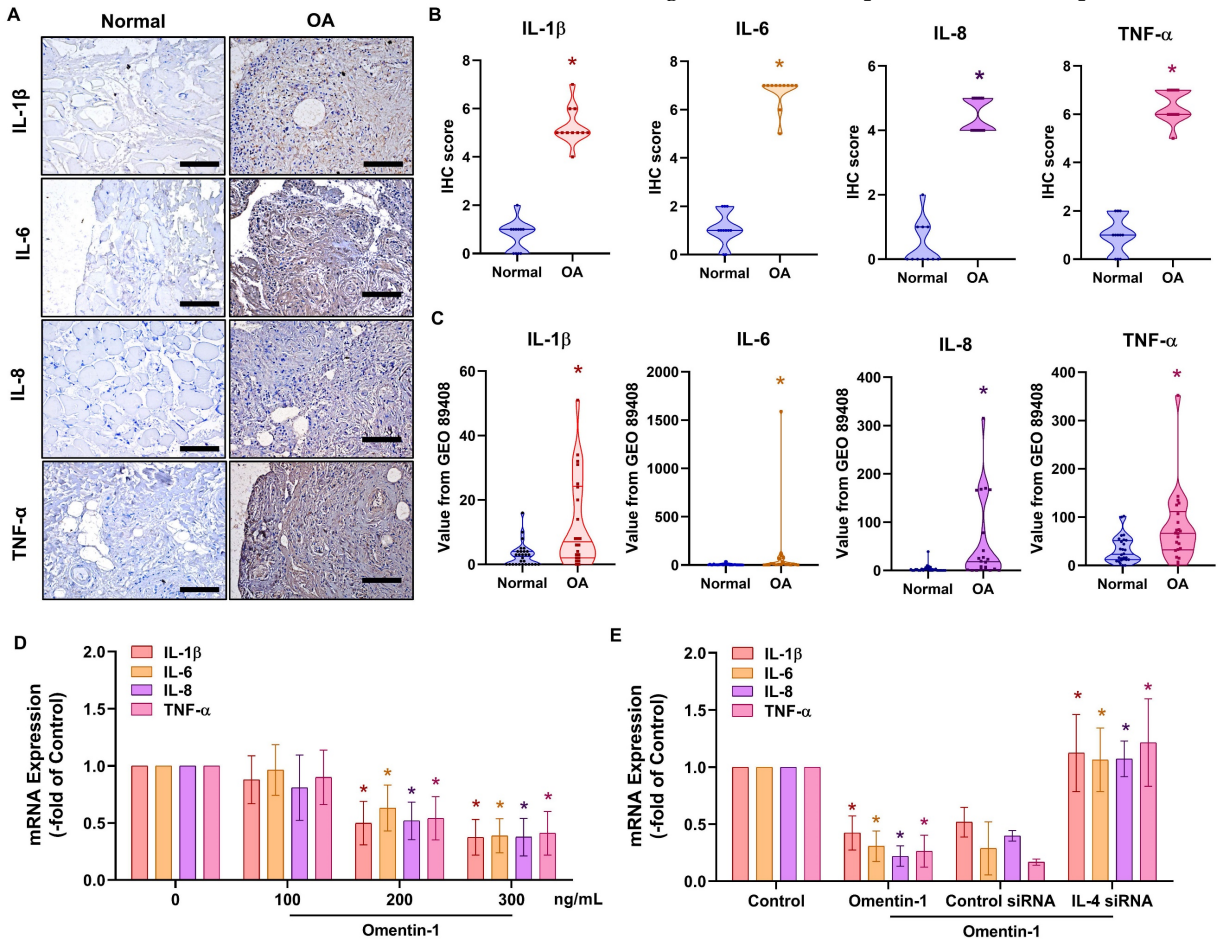
** Omentin-1 inhibited the production of pro-inflammatory cytokines by upregulating IL-4 expression.** (A and B) IHC staining showing that IL-1β, IL-6, IL-8, and TNF-α were higher in human OA synovial tissue than in healthy tissue. Scale bar = 100 µm. (C) Gene expression levels in normal and OA synovial tissue samples retrieved from the GEO dataset. (D and E) qPCR analysis showing the gene expression of OASFs treated with omentin-1 or transfected with IL-4 siRNA prior to stimulation using omentin-1, *n*=6 for each group. * *p*<0.05 vs controls. # *p*<0.05 vs omentin-1-treated group.

Proinflammatory cytokines IL-1β, IL-6, IL-8, and TNF-α play dominant roles in OA pathogenesis 6. IHC results and the GEO database revealed elevated expression levels in OA synovial tissue (Fig. 2A-C). Stimulating OASFs with omentin-1 was shown to suppress IL-1β, IL-6, IL-8, and TNF-α production in a concentration dependent manner (Fig. 2D). Transfection with IL-4 siRNA was also shown to antagonize omentin-1-induced inhibition effects (Fig. 2E), which indicates that omentin-1 increases IL-4-dependent anti-inflammatory responses.

### PI3K, ERK and AMPK signaling pathways are involved in the induced expression of IL-4 by omentin-1 in OASFs

**Figure 3:**
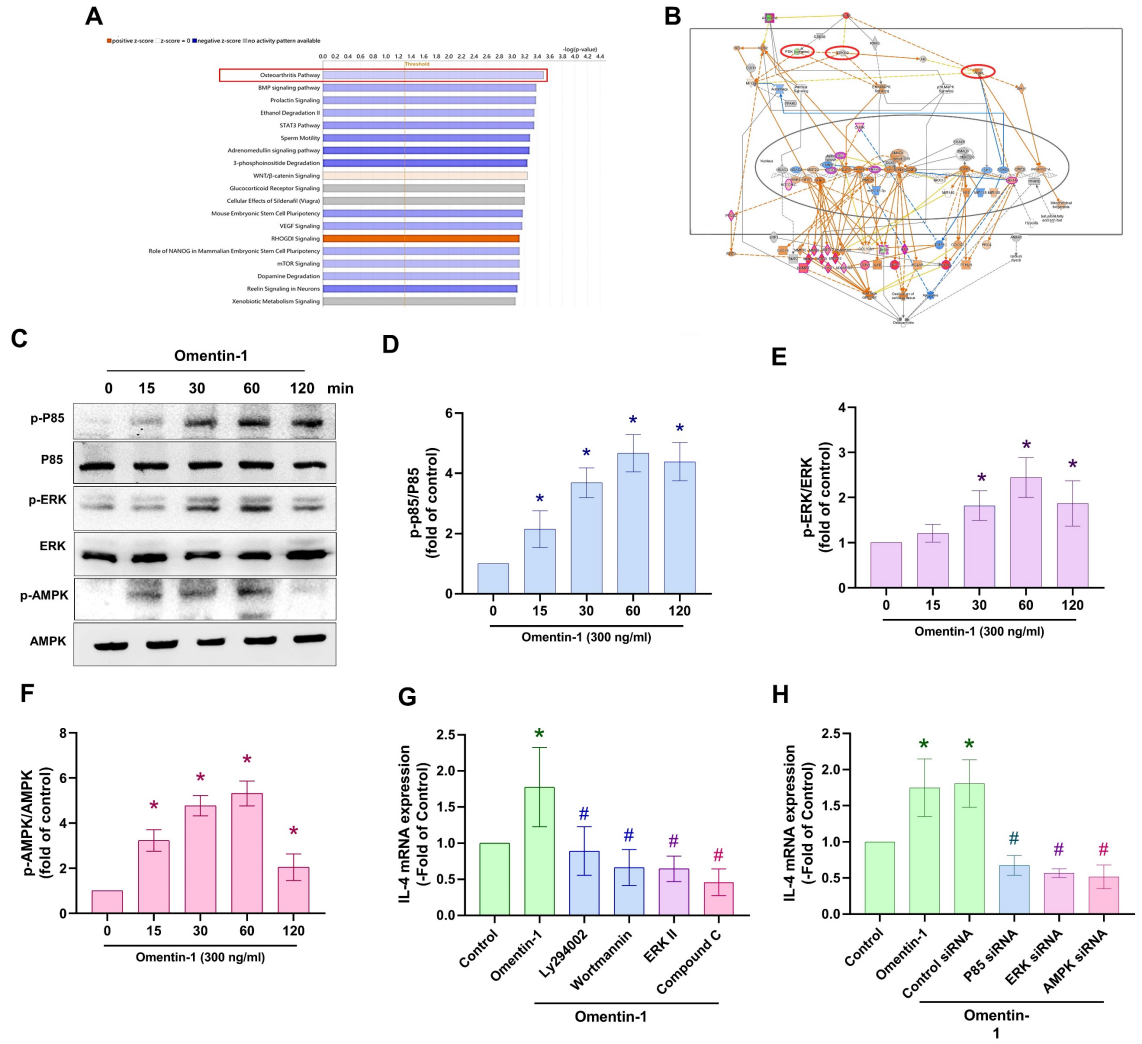
** Omentin-1 promoted IL-4 production in OASFs via the PI3K, ERK, and AMPK pathways.** (A and B) Ingenuity Pathway Analysis (IPA) pathway enrichment figure showing pathways that were significantly upregulated in the GSE82107 database; (C) Western blot analysis showing the phosphorylation of PI3K, ERK, and AMPK in OASFs treated with omentin-1; (D-F) Western blot quantified P85, ERK and AMPK phosphorylation and total protein. (G and H) qPCR analysis showing IL-4 expression in OASFs treated with PI3K inhibitors (Ly294002 and wortmannin), ERK inhibitor (ERK II), and AMPK inhibitor (Compound C) or transfected with indicated siRNA prior to stimulation using omentin-1. * *p*<0.05 vs controls. # *p*<0.05 vs omentin-1-treated group.

We sought to elucidate the mechanism underlying OA by analyzing signaling pathways in the GSE82107 database using Ingenuity Pathway Analysis (IPA) software. Our analysis revealed that the PI3K, ERK, and AMPK signaling pathways were associated with the top-1 signaling pathway; i.e., the OA pathway (Fig. 3A&B). In fact, stimulating OASFs with omentin-1 was shown to facilitate the phosphorylation of PI3K, ERK, and AMPK (Fig. 3C-F). Treating cells with PI3K inhibitors (Ly294002 and wortmannin), an ERK inhibitor (ERK II), or an AMPK inhibitor (Compound C) was shown to block omentin-1-induced IL-4 expression (Fig. 3G). Transfecting OASFs with PI3K, ERK, or AMPK siRNA had similar effects (Fig. 3H). Clearly, omentin-1 enhances IL-4 production in OASFs via the PI3K, ERK, and AMPK signaling pathways.

### Omentin-1 facilitates the IL-4-dependent polarization of macrophages to the M2 phenotype

**Figure 4:**
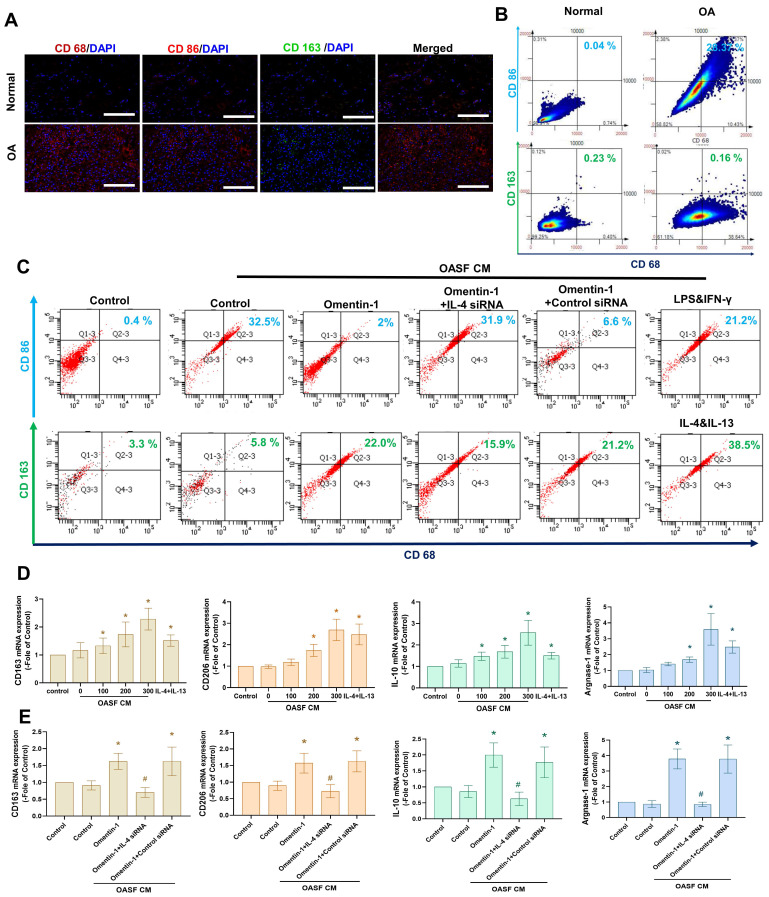
** Omentin-1 enhanced IL-4-dependent polarization of the M2 macrophage.** (A) Immunofluorescence staining for CD68, CD86, and CD163 in synovium tissue samples from normal controls and OA patients. Scale bar = 100 µm. (B) TissueFaxs analysis of fluorescence intensity for macrophage polarization in the ACLT model. (C-E) Flow cytometry analysis and qPCR of THP-1 cells incubated with PMA for 24 h prior to stimulation using LPS+IFN-γ or IL-4+IL-13 or Conditioned Medium (CM) from OASF with or without omentin-1 treatment and IL-4 siRNA transfection for 24 h. * *p*<0.05 vs OASFs group. # *p*<0.05 vs omentin-1-treated OASFs group.

The M2 macrophage phenotype is a major producer of anti-inflammatory cytokines in OA microenvironment 15-17. In the current study, we determined that the expression level of M1 macrophages (but not M2 macrophages) was higher in human OA synovial tissue than in healthy samples, based on double immunofluorescence staining of the synovium using CD68 (a macrophage marker,) CD 86 (an M1 phenotype marker), and CD163 (a M2 phenotype marker) (Fig. 4A). We then sought to determine whether the M2 macrophage contributes to omentin-1/IL-4 axis-mediated anti-inflammatory responses. Incubating M0 macrophages with OASF conditional medium (CM) was shown to promote polarization to M1 macrophages, based on marker CD86, while the effect was reduced when using CM with omentin-1-treated OASFs. Transfecting OASFs with IL-4 siRNA blocked omentin-1-mediated effects (Fig. 4B). Polarization was markedly higher in M0 macrophages incubated with CM containing omentin-1-treated OASFs than in M0 macrophages incubated in CM containing only OASFs, based on the expression level of the M2 marker CD163 (Fig. 4B). M2 macrophage polarization was antagonized by IL-4 siRNA (Fig. 4B). Note that LPS + IFN-γ treatment and IL-4 + IL-13 treatment respectively served as positive controls for M1 and M2 macrophages. In this study, the IgG staining was used as negative control for M1/M2 macrophage (Supplementary Figure S1&S2). The expression of the M2 markers CD163, CD206, IL-10, and arginase 1 (Arg-1) also indicated that CM containing omentin-1-treated OASFs enhanced M2 macrophage polarization, and that transfection with IL-4 siRNA inhibited these effects (Fig. 4C-E). Taken together, these findings indicate that omentin-1 promotes IL-4-dependent M2 macrophage polarization.

### Omentin-1 antagonized ACLT-induced OA *in vivo*

**Figure 5:**
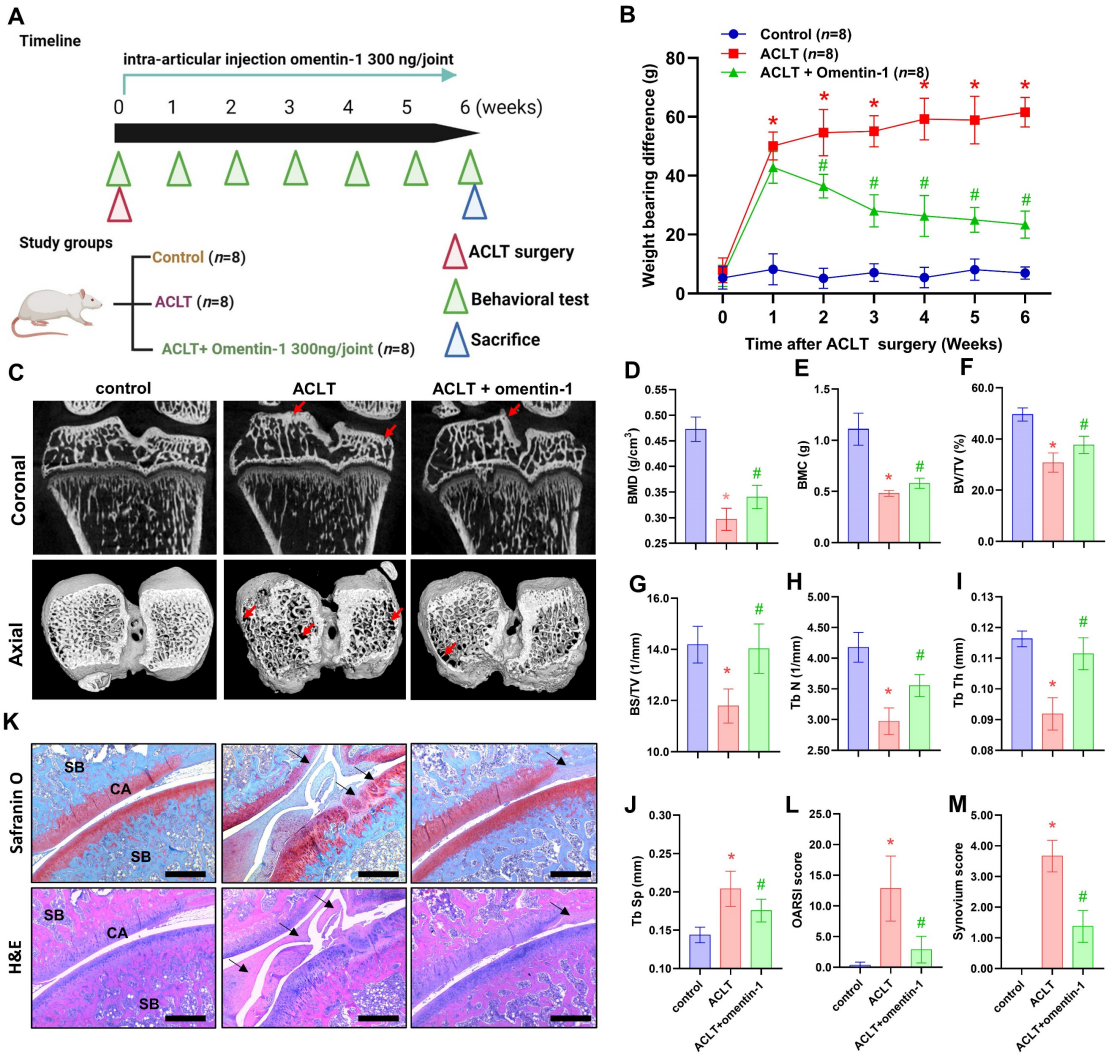
** Omentin-1 antagonized ACLT-induced breakdown of cartilage and bone.** (A) Experiment flow involving ACLT induction and omentin-1 injection; (B) Weight-bearing asymmetry analysis; (C) Photomicrographs showing coronal and axial views of micro-CT images. Red arrow indicates the bone loss; (D-J) Graphic illustrations of BMD, BMC, bone volume, bone surface, trabecular numbers, trabecular bone thickness, and trabecular separation in the indicated groups; (K-M) Histological sections from knees stained with H&E and Safranin-O with corresponding OARSI and synovium scores. Black arrows indicate the cartilage damage or synovial hyperplasia. Scale bar =500 µm. Abbreviations: ST, synovial tissue; SB, subchondral bone; CA, cartilage. * *p*<0.05 vs control group. # *p*<0.05 vs ACLT group.

The potential therapeutic benefits of omentin-1 for OA were assessed using an ACLT-induced OA model, which involved the intra-articular administration of omentin-1 on a daily basis for 6 weeks (Fig. 5A). Articular nociception was evaluated using a dynamic weight bearing apparatus. The ACLT group presented a significant increase in weight bearing capacity between the 1^st^ and 6^th^ weeks after surgery (Fig. 5B); however, the improvement was gradual between the 2^nd^ and 6^th^ weeks after the initiation of treatment with omentin-1 (Fig. 5B). At 6 weeks after surgery, the ACLT group presented bone damage and boss loss in micro-CT images. At the same time point, the ACLT + omentin-1 group presented a significant reversal in the surface area affected by bone wear and bone erosion (Fig. 5C). In micro-CT scan analysis of the trabecular bone, bone mineral density (BMB), bone mineral content (BMC), bone volume (BV/TV), bone surface (BS/TV), trabeculae number (Tb N), and trabeculae thickness (Tb Th) were higher in the ACLT + omentin-1 group than in the ACLT group, while the extent of trabecular separation was lower (Tb Sp) (Fig. 5D-J). Staining using H&E and Safranin-O/Fast Green confirmed that the decrease in OARSI score and synovial inflammation (synovium score) was more pronounced in the ACLT + omentin-1 group than in the ACLT group (Fig. 5K-M).

### Omentin-1 enhanced IL-4 production, anti-inflammatory responses, and expression of the M2 macrophages phenotype *in vivo*

**Figure 6:**
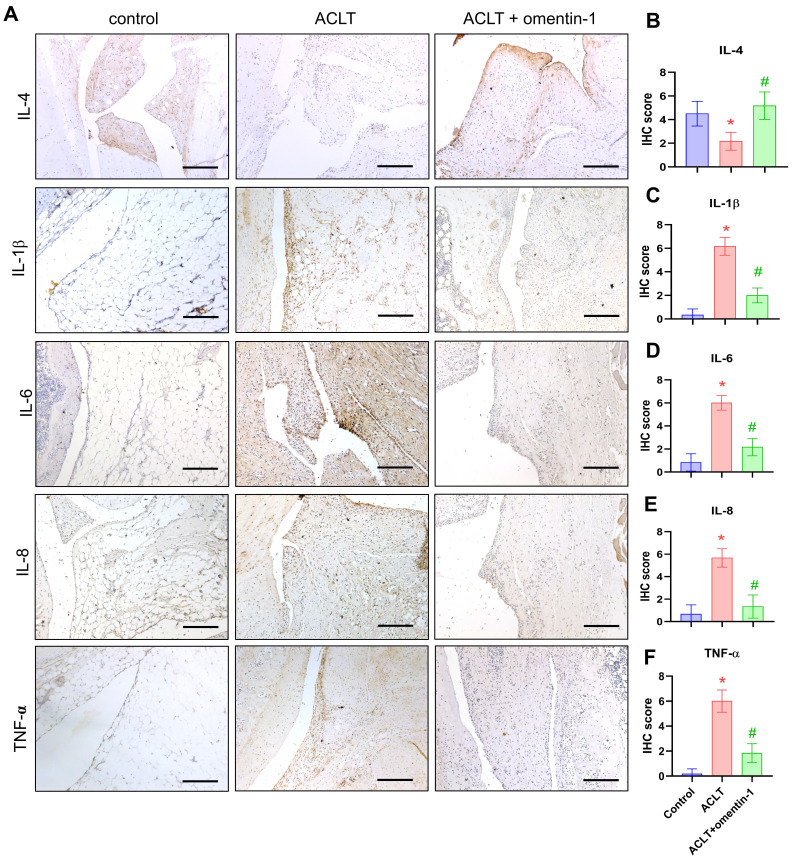
** Omentin-1 increased IL-4 production and reduced the expression of pro-inflammatory cytokines in ACLT model.** (A) Immunohistochemistry (IHC) staining for IL-4, IL-1β, IL-6, IL-8, and TNF-α and (B-F) quantification of IHC scores. Scale bar =100 µm. *n*=6 for each group. * *p*<0.05 vs control group. # *p*<0.05 vs ACLT group.

**Figure 7:**
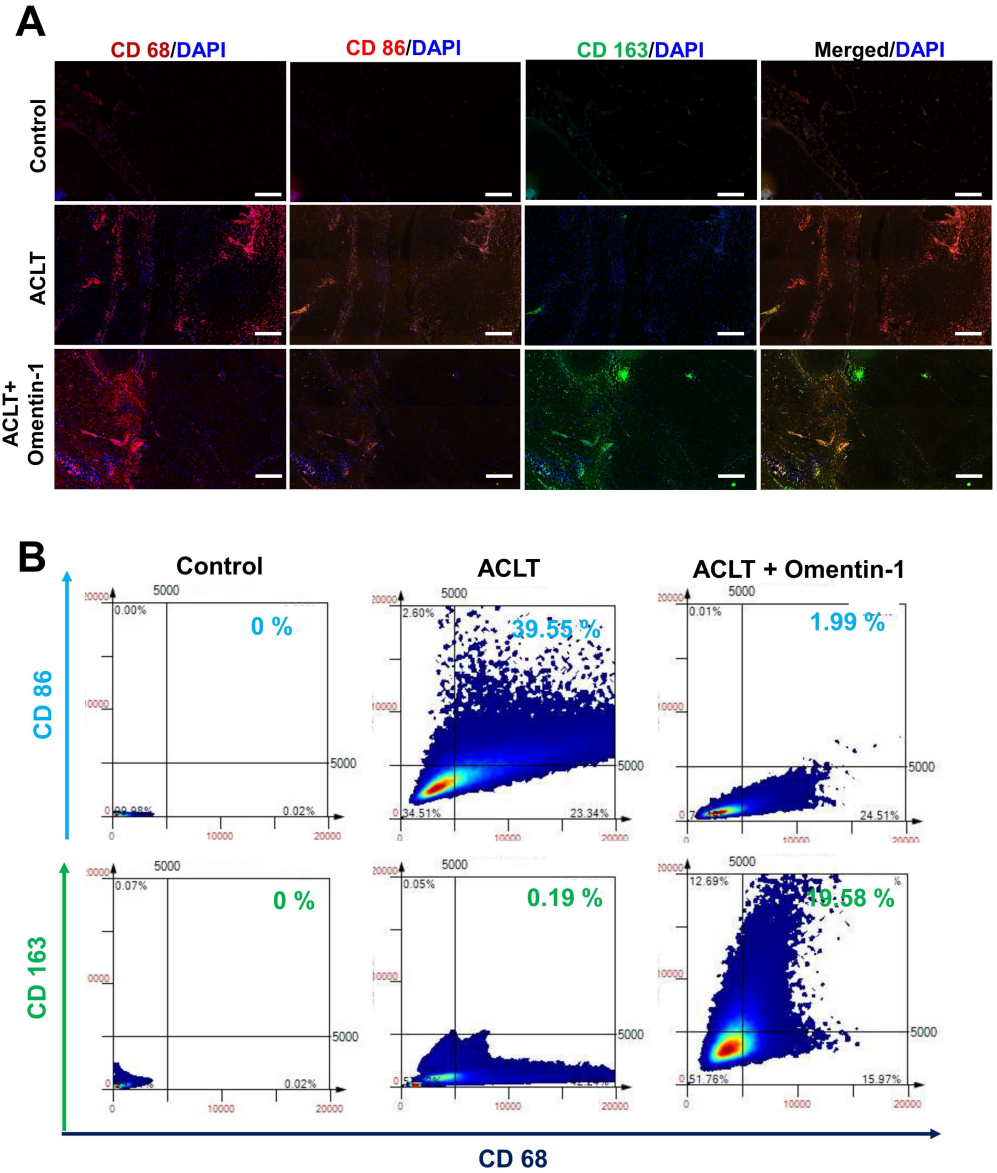
** Omentin-1 treatment increased the number of M2 macrophages in ACLT model.** (A) Immunofluorescence staining for CD68, CD86, and CD163; and (B) TissueFaxs analysis of fluorescence intensity of macrophage polarization in ACLT model. Scale bar =100 µm. * *p*<0.05 vs control group. # *p*<0.05 vs ACLT group.

Compared with samples in the ACLT group, omentin-1 was shown to up-regulate the expression of IL-4 and down-regulate the expression of IL-1β, IL-6, IL-8, and TNF-α (Fig. 6). Immunofluorescence data revealed that ACLT increased the expression of M1 macrophages (Fig. 7). Omentin-1 treatment was shown to down-regulate M1 macrophages levels and up-regulate M2 macrophage levels (Fig. 7). These results indicate that omentin-1 suppressed ACLT-induced OA progression by up-regulating IL-4 production, anti-inflammatory responses, and expression of the M2 macrophage phenotype. In this study, the IgG staining also was used as negative control for M1/M2 macrophage on ACLT group (Supplementary Figure S3).

## Discussion

OA is a chronic degenerative joint disease characterized by the loss of articular cartilage, subchondral bone changes, and synovial inflammation 32. The synovium, a thin layer of tissue lining the joints, plays a crucial role in the pathogenesis of OA by producing pro-inflammatory cytokines, chemokines, and proteases 33. Synovial inflammation is a key feature of OA and contributes to pain, cartilage damage, and joint stiffness 34. Most current treatments for OA focus on alleviating pain, while largely disregarding the underlying mechanisms. Preclinical studies have identified several potential therapeutic targets for the treatment of OA 35. Two promising approaches to treating OA include blocking inflammatory cytokines and developing anti-inflammatory therapeutics 36. This study presents novel evidence suggesting that omentin-1 has anti-inflammatory properties, which could potentially be harnessed to mitigate the effects of OA. This makes omentin-1 is a novel molecular target for the development of OA therapies.

The process of inflammation involves the release of various adipokines, such as leptin, adiponectin, resistin, visfatin, chemerin-1, vaspin, omentin, meteorin-like, retinol binding protein-4, and irisin by adipose tissue, which can exacerbate synovium and chondrocyte degeneration and breakdown of the extracellular matrix 37. Clinical studies have also reported a positive correlation between the levels of some synovial adipokines and the activity index of elderly patients with OA affecting the knees 38-41. Omentin-1 is a novel adipokine that plays various protective roles in maintaining metabolic processes and insulin sensitivity. Omentin-1 also exerts anti-inflammatory, anti-atherosclerotic, and cardiovascular protective effects 20, 42-44. The observed anti-inflammatory effect of omentin-1 is in line with the inverse correlation between synovial omentin-1 levels and radiographic severity of OA reported in previous studies 45. In the current study, our results from the GEO dataset and clinical samples revealed that omentin-1 levels are significantly lower in OA patients than in normal controls. Treating OASFs with omentin-1 was shown to decrease the production of pro-inflammatory cytokines. Omentin-1 was also shown to block the ACLT-induced breakdown of cartilage and bone *in vivo*. We also obtained evidence that omentin-1 is an anti-inflammatory adipokine applicable to the treatment of OA.

Studies have demonstrated that IL-4 produced by T-cells can decrease the production of cytokines that possess cytostatic activity for tumor cells, while inhibiting the secretion of IL-1β, TNF-α, and IL-6 by human monocytes 10. Note that IL-4 protects the body against OA by creating an immunomodulatory microenvironment in which joint-resident macrophages polarize toward an M2 phenotype to facilitate the clearance of pro-inflammatory debris 46. IL-4 also helps to maintain activity in the osteoclasts of subchondral bone at homeostatic level, which can be beneficial for the treatment of OA 46. Taken together, these results indicate that IL-4 could potentially protect the body against OA. In the current study, we determined that omentin-1 promoted IL-4 production *in vitro* and *in vivo*. The genetic inhibition of IL-4 was shown to antagonize the production of omentin-1-inhibited pro-inflammatory cytokines. IL-4 siRNA also blocked the promotion of M2 macrophage polarization by omentin-1-treated OASFs. Thus, it appears that omentin-1 facilitates IL-4-dependent anti-inflammatory responses as well as M2 macrophage polarization in OA.

Previous investigations have shown that omentin-1 ameliorates atherosclerosis, endothelial dysfunction, and inflammation via AMPK, PI3K and MAPK signal pathways 43, 47, 48. These pathways play the important roles in inflammatory response, for example the PI3K recruits and activates innate immune cells, the ERK mediates proliferation, growth and differentiation in inflammatory cells, and the AMPK enhances autophagy, inflammation, immunity and osteoclast differentiation 49-51. Our IPA analysis results revealed that omentin-1 associated PI3K, ERK and AMPK pathways in OA progression. In addition, inhibition of PI3K, ERK and AMPK signaling by using pharmacological agents or genetic siRNAs reversed omentin-1 enhanced IL-4 production in OASFs. Furthermore, stimulation of omentin-1 facilitates PI3K, ERK and AMPK phosphorylation in OASFs. Thus, it appears that omentin-1 facilitates IL-4 synthesis in OASFs through PI3K, ERK and AMPK pathways.

Depending on the local microenvironment, resting macrophages (M0) can polarize into various phenotypes, including pro-inflammatory (M1) or anti-inflammatory (M2) phenotypes. Their functions also vary with physiological and pathological conditions 52, 53. Synovial macrophages act as immune cells crucial to the symptomatology and structural progression of OA 54. These activated macrophages are regulated by a variety of stimulators and can polarize into either M1 or M2 subtypes in synovial tissue, synovial fluid, or peripheral blood 55. One study reported that injecting M2 macrophages directly into joints can prevent the formation of osteophytes and stave off damage to cartilage during the development of OA 56, which suggests that M2 macrophages have potential as anti-OA compounds. In the current study, M1 expression (but not M2 expression) was higher in OA patients than in healthy controls. CM containing OASF-treated omentin-1 was shown to polarize M0 macrophages to the M2 phenotype, which further suggests that omentin-1 plays a protective role in the OA microenvironment. Our ACLT model also demonstrated the effects of omentin-1 in promoting the formation of M2 macrophages* in vivo*. This is a clear indication that M2 macrophage polarization contributed the observed omentin-1-regulated anti-inflammatory response in OA. For further application, development of pharmacological or genetic activator of omentin-1 used in OA treatment. Furthermore, our results also show that omentin-1 have the immunomodulatory characteristic on macrophage polarization which can be applied in other inflammatory diseases such inflammatory bowel disease and rheumatic arthritis.

**Figure 8:**
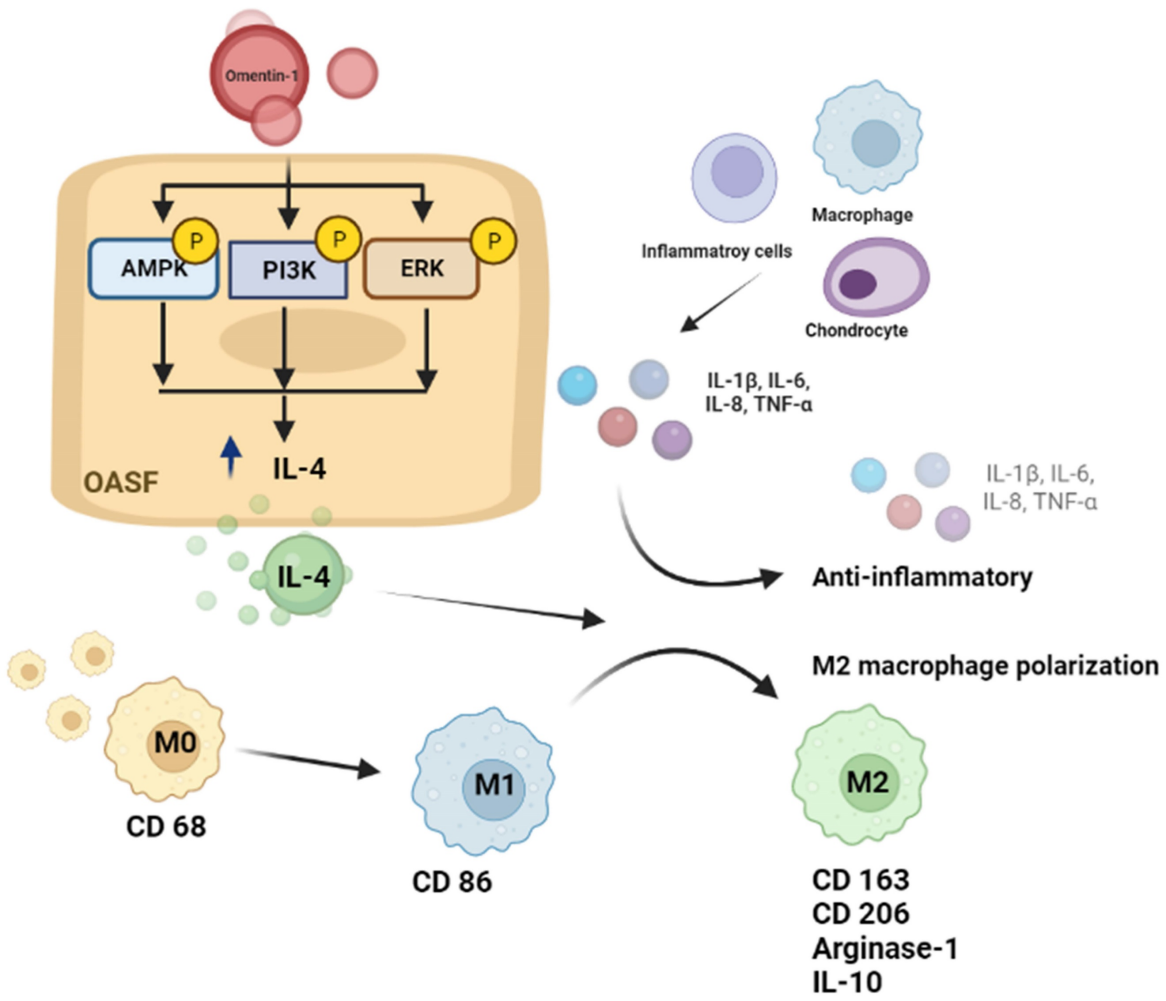
** Schematic diagram illustrating the mechanisms of omentin-1 function in OA.** The schematic diagram summarizes the mechanisms underlying the omentin-1-induced increase in the production of IL-4 in human OASFs via the PI3K, ERK, and AMPK pathways, which inhibited the expression of pro-inflammatory cytokines, while facilitating M2 macrophage polarization and antagonizing OA progression.

In conclusion, we determined that omentin-1 and IL-4 play protective roles during OA development. Omentin-1 facilitates IL-4-depedent anti-inflammatory responses and M2 polarization via the PI3K, ERK, and AMPK pathways in OASFs (Fig. 8). Omentin-1 was also shown to antagonize ACLT-mediated cartilage degradation and bone erosion *in vivo*. Omentin-1 is a strong candidate for the development of novel therapeutic treatments for OA.

## Supplementary Material

Supplementary figures and table.
