# Supplementary material for: Omentin-1 ameliorates the progress of osteoarthritis by promoting IL-4-dependent anti-inflammatory responses and M2 macrophage polarization
Source: Int J Biol Sci. 2023 Oct 16;19(16):5275–89. doi: 10.7150/ijbs.86701 (PMC10620827; doi:10.7150/ijbs.86701)
Supplement: Supplementary file 1 — Supplementary figures and table. [file ijbsv19p5275s1.pdf]

Omentin-1 ameliorates the progress of osteoarthritis by promoting IL-4-dependent anti-inflammatory responses and M2 macrophage polarization

Chih-Yuan Ko, Yen-You Lin, David Achudhan, Jun-Way Chang, Shan-Chi Liu, Chao-Yang Lai, Yuan-Li Huang, Chun-Hao Tsai, Yi-Chin Fong, Hsien-Te Chen, Kun-Tsan Lee, Chien-Chung Huang, Ting-Kuo Chang and Chih-Hsin Tang

This file includes:

Supplementary data

Supplementary Table

Supplementary data

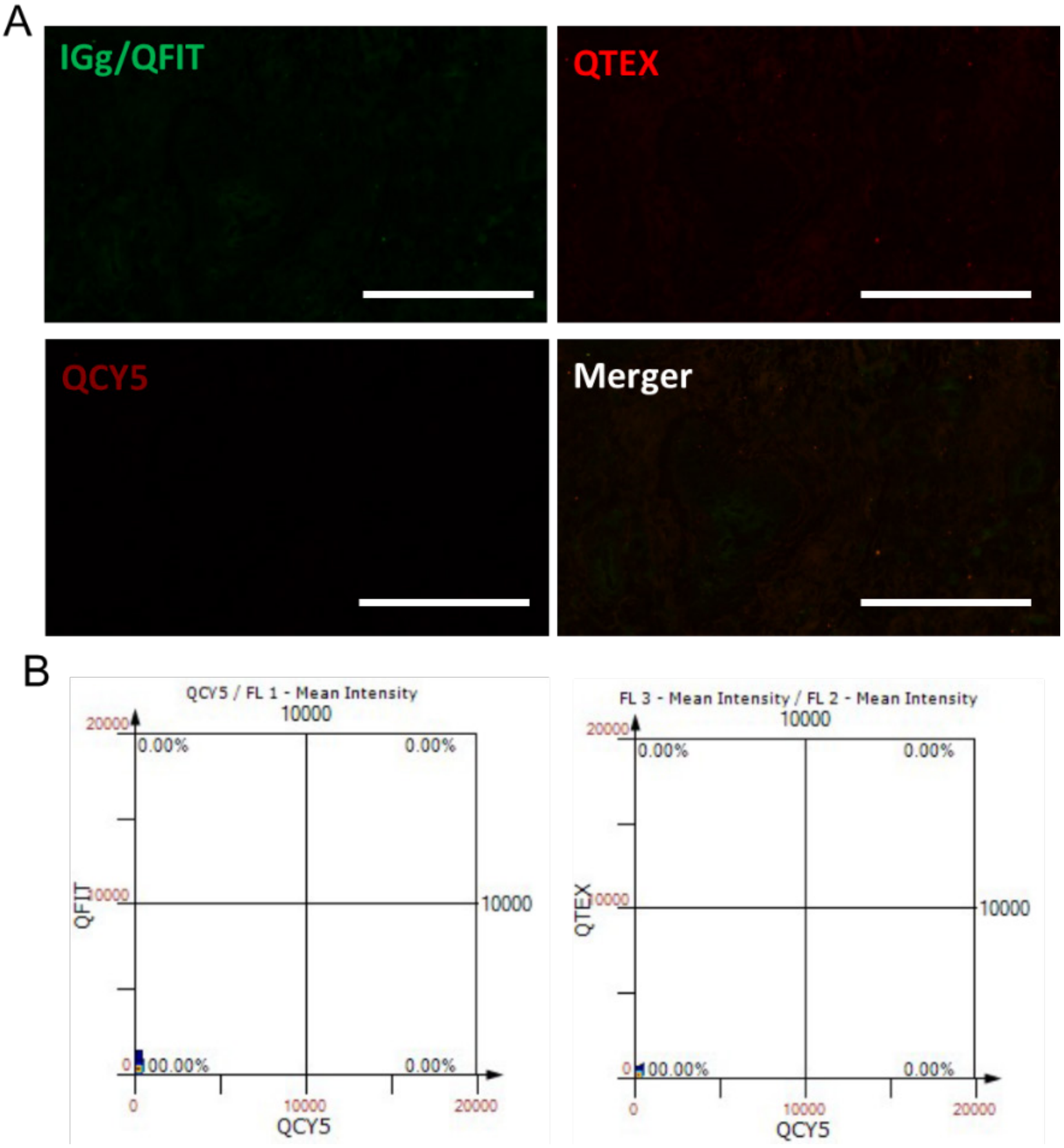

Supplementary Figure S1. (A) Immunofluorescence staining for IgG in synovial tissue samples from OA patients. (B) TissueFaxs analysis of fluorescence intensity for fluorescein isothiocyanate (QFIT), Texas red (QTEX), and Cyanine dye cy5 (QCY5).

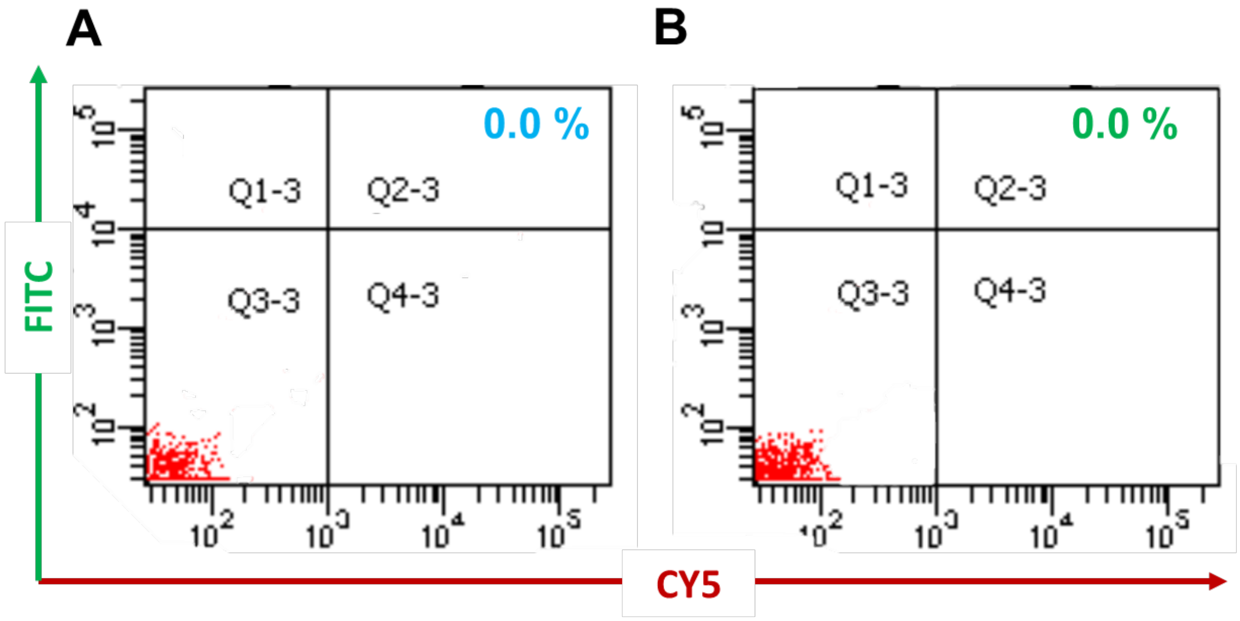

Supplementary Figure S2. Flow cytometry analysis of THP-1 cells incubated with PMA for 24 hours prior to stimulation using conditioned medium (CM) from OASF with (A) or without (B) omentin-1 treatment for 24 hours. Fluorescein isothiocyanate (FITC), and Cyanine dye cy5 (CY5).

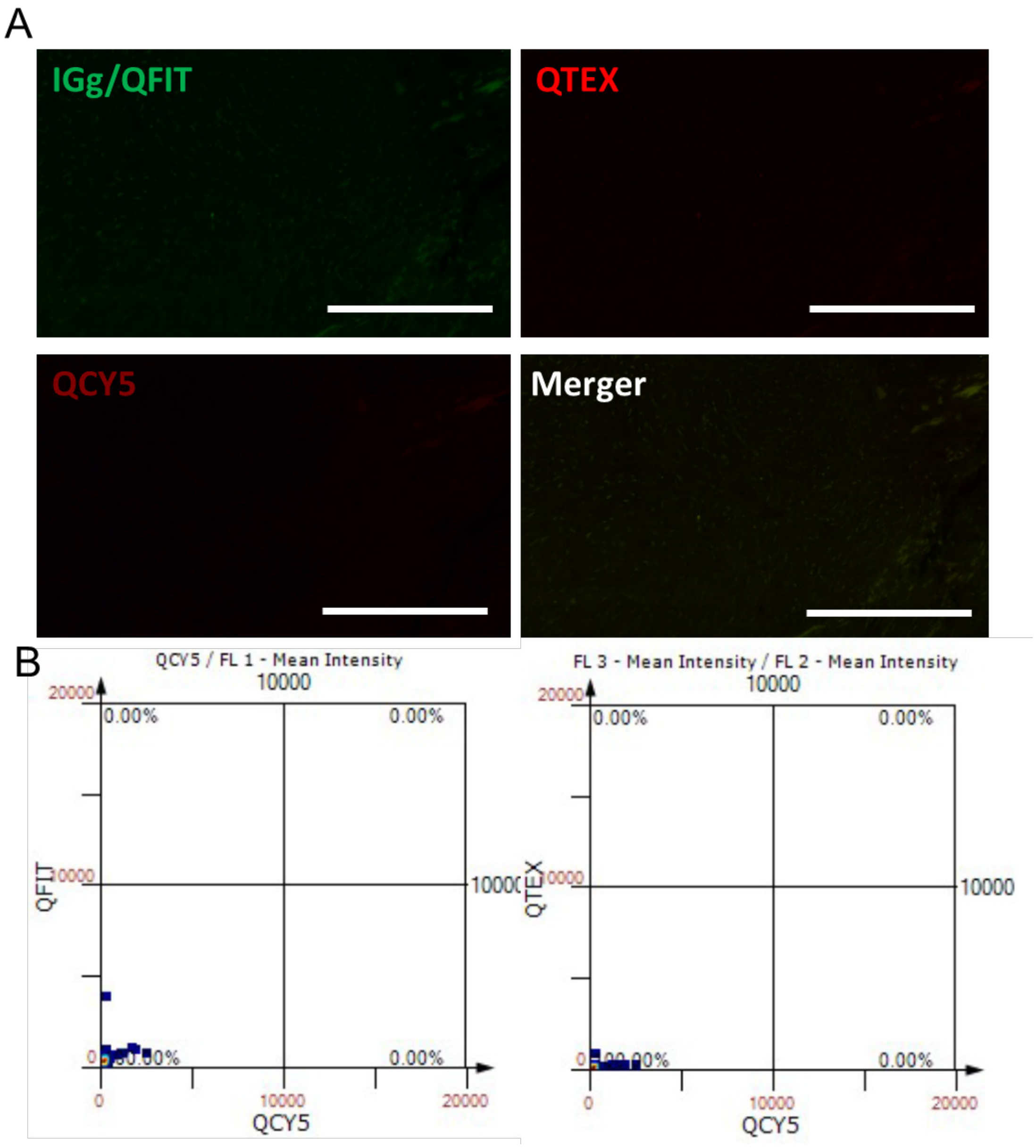

Supplementary Figure S3. (A) Immunofluorescence staining for IgG in synovial tissue samples from ACLT rats. (B) TissueFaxs analysis of fluorescence intensity for Fluorescein isothiocyanate (QFIT), Texas red (QTEX), and Cyanine dye cy5 (QCY5).

Supplementary Table S1. Primers used in this study.

Gene	Forward	Reverse
Arginase 1	GTGGAAACTTGCATGGACAAC	AATCCTGGCACATCGGGAATC
CD163	TTTGTCAACTTGAGTCCCTTCAC	TCCCGCTACACTTGTTTTCAC
CD206	GGGTTGCTATCACTCTCTATGC	TTTCTTGTCTGTTGCCGTAGTT
IL-4	CGAGTTGACCGTAACAGACAT	CGTCTTTAGCCTTTCCAAGAAG
IL-1β	ATGATGGCTTATTACAGTGGCAA	GTCGGAGATTCGTAGCTGGA
IL-6	AGACAGCCACTCACCTCTTCAG	TTCTGCCAGTGCCTCTTTGCTG
IL-8	CTGCGCCAACACAGAAATTATTGTA	TTCACTGGCATCTTCACTGATTCTT
IL-10	GACTTTAAGGGTTACCTGGGTTG	TCACATGCGCCTTGATGTCTG-3
IL-13	ACGGTCATTGCTCTCACTTGCC	CTGTCAGGTTGATGCTCCATACC
GAPDH	AATGGACAACTGGTCGTGGA	CCCTCCAGGGATCTGTTTG
TNF-α	CCTCTCTCTAATCAGCCCTCTG	GAGGACCTGGGAGTAGATGAG
